# Accuracy and Reproducibility of Handheld 3D Ultrasound Versus Conventional 2D Ultrasound for Urinary Bladder Volume Measurement: A Prospective Comparative Study

**DOI:** 10.3390/diagnostics15172229

**Published:** 2025-09-03

**Authors:** Abdulrahman M. Alfuraih, Saleh K. S. Alkuwileet, Abdulmalik K. Alhoysin, Abdulmajed S. Alhawwashi, Abdullah I. Aldakan, Fahad K. Alotaibi, Mohammed J. Alsaadi

**Affiliations:** Radiology and Medical Imaging Department, College of Applied Medical Sciences, Prince Sattam bin Abdulaziz University, Al-Kharj 16278, Saudi Arabia441050794@std.psau.edu.sa (A.S.A.); 441051198@std.psau.edu.sa (A.I.A.); 441051139@std.psau.edu.sa (F.K.A.); m.alsaadi@psau.edu.sa (M.J.A.)

**Keywords:** urinary bladder volume, point-of-care ultrasound, 3D ultrasound, handheld ultrasound device, bladder volume measurement, post-void residual urine, measurement accuracy, inter-operator reproducibility, bedside ultrasound, bladder scanner

## Abstract

**Background/Objectives:** Accurate urinary bladder (UB) volume measurement is essential for diagnosing urinary retention, evaluating post-void residuals, and guiding catheterization decisions. Conventional 2D ultrasound and automated non-visual bladder scanners can be limited by operator variability and systematic errors. Three-dimensional (3D) ultrasound may improve accuracy and reproducibility, but data on handheld, semi-automated devices remain scarce. This study aimed to compare the accuracy, reproducibility, and feasibility of a handheld 3D ultrasound device versus conventional 2D ultrasound for UB volume estimation, using measured voided volume as the reference standard. **Methods:** Fifty-three healthy male volunteers (mean age 19.6 ± 2.0 years) underwent bladder volume assessment by two novice operators using both methods: 2D ultrasound (manual caliper-based) and handheld 3D ultrasound device (Butterfly iQ). Each operator performed two repeated measurements per method. True voided volume was recorded immediately after scanning. Accuracy was assessed using median differences, percentage error, and Bland–Altman analysis. Intra- and inter-operator reproducibility were evaluated with intraclass correlation coefficients (ICC). **Results:** Both methods significantly underestimated bladder volume (*p* < 0.001). The 3D method demonstrated higher accuracy, with a median percentage error of −11.2% to −12.0%, versus −27.6% to −36.7% for 2D. The mean bias ranged from −64.9 mL to −72.3 mL for 3D, compared to −137.4 mL to −191.6 mL for 2D. Intra-operator reproducibility was excellent for all methods (ICC > 0.96). Inter-operator agreement was higher for 3D (ICC = 0.977; bias 7.3 mL) than for 2D (ICC = 0.927; bias −54.2 mL). All scans were completed successfully; however, the 3D device occasionally faced technical errors in large bladder volumes. **Conclusions:** Handheld 3D ultrasound yielded greater accuracy and inter-operator consistency than conventional 2D ultrasound in healthy adults, with minimal operator input. Both methods underestimated true volume, indicating the need for clinical consideration when interpreting measurements. These findings support broader clinical adoption of handheld 3D ultrasound, particularly in settings with variable sonographic expertise, while highlighting the need for validation in elderly and pathological populations.

## 1. Introduction

Accurate measurement of urinary bladder (UB) volume is critical in patient care in numerous clinical contexts, including the assessment of urinary retention, evaluation of post-void residual (PVR) urine, and guidance for catheterization [1,2]. Incorrect estimation may lead to unnecessary catheterization or missed diagnoses, both of which have substantial risks such as urinary tract infection, bladder overdistension, and compromised patient outcomes [3].

Multiple techniques are available for UB volume assessment, ranging from catheterization (considered the gold standard but invasive) to non-invasive imaging modalities such as ultrasonography. Among these, point-of-care ultrasound (POCUS) has emerged as a preferred method, offering rapid, bedside assessment with minimal discomfort [1]. Conventional approaches include the two-dimensional (2D) ultrasound, where bladder dimensions are measured in orthogonal planes and volume is estimated via geometric formulas. Additionally, automated ultrasound bladder scanners are widely used to estimate urinary bladder volume without providing direct visualization of the bladder. These devices typically present only a numerical volume reading or a basic schematic representation, rather than a true ultrasound image. As a result, anechoic structures such as abdominal or pelvic fluid collections can potentially distort the measurements [4]. Multiple studies have shown that the reproducibility and accuracy of such non-visual scanners are inferior to that of imaging-based 2D scanning. This has been observed in specific patient groups, including children [5,6], postpartum women [7], women undergoing uroflowmetry [8], and cases with complicated conditions such as pelvic organ prolapse [9].

Three-dimensional (3D) ultrasound is an alternative method that could enable more accurate volumetric reconstruction and reduce reliance on manual caliper placement [10,11]. Comparative studies have consistently demonstrated that 3D methods improve both accuracy and inter-operator reproducibility over 2D techniques [12]. However, these studies often involve cart-based or semi-automated 3D systems that require significant operator expertise and specific reconstruction software to determine bladder boundaries [11,12,13]. Recent advances in handheld ultrasound technology have introduced compact, portable devices capable of performing fully automated 3D bladder volume measurements with minimal user input. Early evaluations suggest these devices may deliver clinically acceptable accuracy while requiring little operator training [14,15,16,17,18]. Despite these promising findings, existing studies often involve small sample sizes and lack operator reproducibility testing. Emerging trends in urology also include a growing interest in complementary and alternative medicine approaches for managing bladder health and capacity, underscoring the need for accurate volumetric assessment tools to evaluate their impacts [19].

The present study aimed to directly compare bladder volume measurement accuracy and reproducibility between the conventional 2D ultrasound method and a semi-automated 3D method on a handheld ultrasound. We hypothesized that the 3D method would demonstrate improved accuracy and inter-operator reproducibility over the conventional 2D method, while maintaining clinical feasibility.

## 2. Materials and Methods

### 2.1. Study Design

This cross-sectional study included healthy male medical college students at Prince Sattam bin Abdulaziz University recruited through convenience sampling using personal relationships and word-of-mouth to obtain the largest feasible sample. Volunteers were eligible if they were over 18 years old and had no previous history of urinary bladder operations. The study was approved by the university ethics committee (approval number: REC-HSD-116-224), and informed consent was obtained from all participants prior to enrollment.

### 2.2. Acquisition Methods

The study compared two sonographic methods for bladder volume estimation accuracy and agreement. The first method (2D method) employed traditional 2D bladder volume measurement by calculating height, width, and depth dimensions using the HI VISION Avius ultrasound system (Hitachi Medical Corporation, Tokyo, Japan) equipped with an EUP-C715 Abdominal Convex transducer operating at 1–5 MHz frequency range. This device automatically calculates bladder volume using the built-in calculation tool that assumes a spheroidal shape according to the formula:

$$Volume=\frac{\pi }{6}\left(height\times width\;\times depth\right)$$

The second method (3D method) utilized the handheld Butterfly iQ ultrasound device (Butterfly Network Inc., Guilford, CT, USA), which employs Capacitive Micromachined Ultrasonic Transducers (CMUT) operating at 1–10 MHz frequency range [20,21]. This device acquires 3D bladder volumes by electronically sweeping the ultrasound beam across the bladder area using its proprietary 3D software: “Butterfly Auto Bladder Volume Tool”. The manufacturer reports a volume accuracy of ±7.5% using this tool [22]. The device was connected to a 12.9″ tablet (iPad Pro, Apple Inc., Cupertino, CA, USA) for image control and acquisition.

### 2.3. Study Protocol

Two novice operators (S.K.S.A and A.K.A) with six months of ultrasound imaging training acquired all measurements using both systems. We intentionally chose a small number of operators to simulate the typical users of handheld devices and to minimize bladder filling during repeated scans, which can confound accuracy assessments. Nevertheless, the operators had sufficient training on the standard 2D method but no previous experience with the 3D method. Hence, a single simple demo on how to acquire valid results was demonstrated to them by an expert consultant in ultrasonography (A.M.A). A third research team member (A.I.A) recorded the bladder volume measurements while maintaining operator blinding to prevent measurement bias.

Participants were instructed to drink 1 L of water at least 30 min prior to their scheduled appointments to ensure adequate bladder filling. They were then asked to inform a research team member of a full bladder sensation. When this is noted, the first scan began after confirming the presence of at least 150 mL in the bladder using the conventional 2D method.

The measurement protocol involved Operator A performing two consecutive bladder volume measurements using the standard 2D method, immediately followed by two consecutive measurements using the 3D method. Operator B then repeated this identical sequence. Each scan was performed independently, and the transducer was removed between repeated measurements to simulate separate acquisitions. Pilot testing with three participants confirmed that repeated acquisitions could be completed within less than one and a half minutes. Although the precise interval was not recorded during the main study, the short acquisition time was considered insufficient for meaningful bladder filling to occur. All measurements were recorded by an independent research team member, with operators remaining unaware of their values throughout the procedure.

Following completion of all ultrasound measurements, participants completely voided their bladders into a urine collection container. The total voided volume was determined by weighing it using an assumed urine density of 1.0 g/mL, serving as the reference standard for accuracy assessment. A pilot study involving three participants was conducted to verify protocol feasibility and estimate examination duration prior to the main study.

### 2.4. Statistical Methods

Statistical analysis was conducted to compare the accuracy, reproducibility and agreement of bladder volume measurements obtained using the different techniques. All statistical analyses were performed using JASP software (version 0.95), except where mentioned [23]. Given that the data did not meet the assumption of normality, as confirmed by histograms and the Kolmogorov–Smirnov test (*p* < 0.05), non-parametric tests were employed where appropriate.

Descriptive statistics, including median, interquartile range (IQR), and mean differences ± standard deviation were calculated for bladder volume measurements obtained by each operator using both techniques. Bootstrap methods were used to calculate 95% confidence intervals (CI) for medians using SPSS (version 28).

Accuracy was evaluated by comparing ultrasound measurements to the reference standard (measured voided volume). Absolute and percentage differences were calculated for each method. Systematic bias was assessed using Wilcoxon signed-rank tests to determine if median differences significantly differed from zero. Bland–Altman analysis was performed to assess agreement between each ultrasound method and the reference standard, calculating bias (mean difference), limits of agreement (mean ± 1.96 × SD), and their respective 95% confidence intervals.

Intra-operator and inter-operator reproducibility were assessed using Intraclass Correlation Coefficients (ICCs) (two-way mixed effects, absolute agreement, single rater/measurement) with 95% confidence intervals. ICC values were interpreted as follows: <0.5 = poor, 0.5–0.75 = moderate, 0.75–0.9 = good, and >0.9 = excellent reliability. Agreement between the 2D and 3D methods was assessed both within (intra) and across (inter) operators using ICC and Bland–Altman analysis. For intra-operator comparisons, a two-way mixed-effects, absolute agreement ICC was used. For inter-operator inter-method comparisons, a two-way random-effects ICC was used to reflect the random pairing of different operators and methods. Bland–Altman plots were constructed for all reproducibility and agreement assessments, including the mean difference (bias) and 95% limits of agreement (LoA).

This study was designed as an exploratory methods-comparison with an emphasis on estimation (bias, limits of agreement, and ICC) rather than null-hypothesis testing; therefore, no formal a priori sample size calculation was performed. We targeted a sample of approximately 50 participants based on feasibility and to achieve acceptable precision for Bland–Altman limits of agreement and ICC estimates. The final sample (*n* = 53) yielded narrow 95% confidence intervals for both bias/LoA and ICCs. As a reference, *n* = 53 provides >90% power at α = 0.05 to detect a paired difference of ~0.4 SD.

## 3. Results

A total of 53 participants were enrolled in this study. The volunteers had a mean age of 19.6 ± 2.0 years (range: 18–26 years), reflecting a young adult cohort. Participants had a mean height of 173.0 ± 5.7 cm (range: 162.0–191.0 cm) and mean weight of 84.0 ± 21.7 kg (range: 48.1–136.8 kg). The mean body mass index was 28.1 ± 7.3 kg/m^2^ (range: 16.2–50.3 kg/m^2^), indicating a diverse range of body habitus from underweight to obese categories.

### 3.1. Accuracy Assessment

Urinary bladder volume estimation was compared across two methods—standard ultrasound and 3D ultrasound (Butterfly iQ)—performed by two different operators. The median true voided volume was 485.0 mL (95% CI: 441.0–580.0; IQR: 329.4 mL), demonstrating the wide range of bladder volumes in our study population. All ultrasound methods systematically underestimated bladder volume compared to the reference standard of measured voided volume (Table 1).

**Figure 1:**
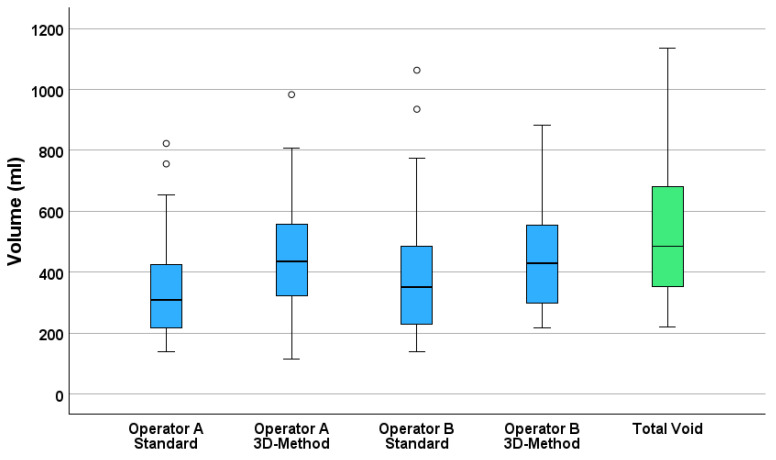
Boxplots of the different urinary bladder volume estimation methods compared to the total measured voided volume (green box).

Additionally, all methods significantly differed from the true voided volume based on Wilcoxon signed-rank tests (all *p* < 0.001). Figure 1 presents boxplots comparing all four estimation methods to the measured voided volume, illustrating the systematic underestimation by the standard methods and the improved estimations of 3D measurements to the voided volume.

Operator A’s standard method significantly underestimated bladder volume with a median estimate of 309.7 mL (95% CI: 257.3–352.0), yielding a mean difference of −191.6 mL (SD = 88.3) and a relative error of −36.7%. In contrast, the 3D method produced higher accuracy, with a median estimate of 436.0 mL (95% CI: 394.5–524.0), corresponding to a smaller mean difference of −64.9 mL (SD = 83.7) and −11.2% error.

Operator B demonstrated similar trends. The standard method yielded a median estimate of 349.8 mL (95% CI: 306.8–403.4), with a mean difference of −137.4 mL (SD = 69.4) and −27.6% error. The 3D method had a median estimate of 430.0 mL (95% CI: 381.0–520.5), a mean difference of −72.3 mL (SD = 90.6), and −12.0% error.

**Figure 2:**
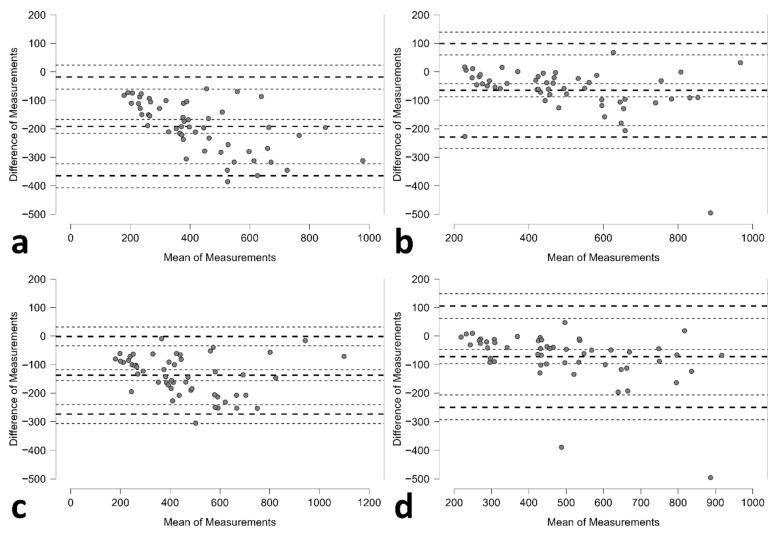
Bland–Altman plots illustrating the agreement between estimated and true voided urinary bladder volumes for each method. The plots show (**a**) Operator A—standard method vs. voided volume, (**b**) Operator A—3D method vs. voided volume, (**c**) Operator B—standard method vs. voided volume, and (**d**) Operator B—3D method vs. voided volume. Each plot displays the mean difference (bias) and the 95% limits of agreement (±1.96 SD), highlighting the systematic underestimation across all methods and a relatively better agreement for the 3D method.

Bland–Altman analysis revealed distinct agreement patterns between methods (Table 2; Figure 2). The standard method demonstrated tighter limits of agreement but with substantial negative bias; whilst the 3D method exhibited wider limits of agreement but smaller systematic bias.

### 3.2. Operators’ Reproducibility

After confirming normal distribution of the differences between repeated measurements, reproducibility was assessed using intraclass correlation coefficients (ICCs) and Bland–Altman analysis (Table 3; Figure S1). Intra-operator reproducibility was excellent for both operators across both methods, with ICC values exceeding 0.96. Operator A showed near-identical reproducibility for the standard (ICC = 0.977) and 3D (ICC = 0.976) techniques, while Operator B showed slightly higher reproducibility with the 3D method (ICC = 0.983) compared to the standard method (ICC = 0.962).

Bland–Altman analyses revealed minimal mean differences between repeated scans for both operators. The 3D method showed a near-zero bias for Operator A (−0.3 mL) and a small positive bias for Operator B (5.8 mL), indicating minimal systematic error. Limits of agreement were narrower for the 3D method than for the standard method, suggesting greater consistency.

Inter-operator agreement was also strong, with higher ICC for the 3D method (0.977) compared to the standard method (0.927). While the standard method showed a notable negative bias (−54.2 mL) between operators, the 3D method demonstrated a much smaller and more symmetric mean difference (7.3 mL), reinforcing its potential for more reliable cross-operator use.

### 3.3. Methods Agreement

The agreement between the standard and 3D ultrasound methods was assessed within and across operators using intraclass correlation coefficients (ICCs) and Bland–Altman analysis (Table 4; Figure S2). Within-operator agreement was good, with ICC values of 0.798 for Operator A and 0.890 for Operator B. Despite this, Bland–Altman plots showed considerable mean differences between the two methods, particularly for Operator A, who exhibited a bias of −126.7 mL (95% CI: −152.5 to −100.9) with wide limits of agreement (−310.3 to 56.9 mL). Operator B demonstrated a smaller bias of −65.2 mL (95% CI: −93.0 to −37.4), but agreement limits remained relatively broad (−262.8 to 132.5 mL).

Cross-operator comparisons between different methods also had some variability. Agreement between Operator A’s standard method and Operator B’s 3D method yielded an ICC of 0.735 and a bias of −119.4 mL, while the reverse comparison of Operator A’s 3D method versus Operator B’s standard method had the highest inter-method ICC (0.901) and a lower bias of 72.5 mL. Notably, the limits of agreement in all comparisons were relatively wide, indicating variability in individual measurements despite good overall correlation. These findings suggest that while 3D ultrasound shows better consistency across operators, considerable variability still exists between methods.

### 3.4. Feasibility Assessment

**Figure 3:**
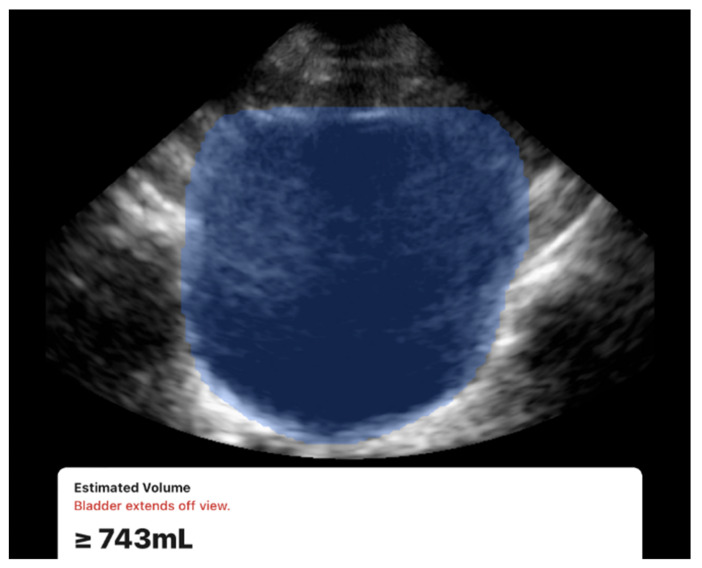
Example of the 3D method scan using the Butterfly iQ device showing the automated bladder volume estimation. Note the “Bladder extends off view” warning which was often observed in cases with large bladder volumes.

**Figure 4:**
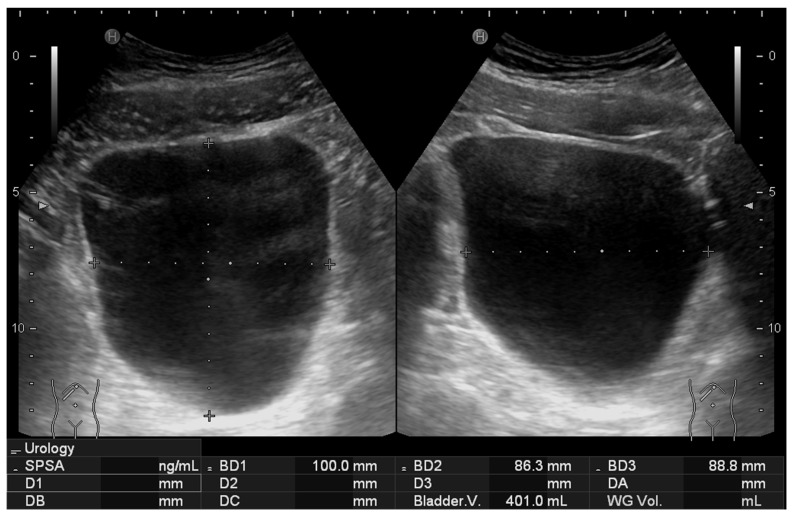
Representative 2D ultrasound image obtained using the standard method, showing the volume estimation at the bottom based on manual caliper measurements.

All scans were successfully completed with no missing data or technical difficulties during data acquisition. Examples of the acquired ultrasound images are presented in Figure 3 and Figure 4. While both devices were usable under all conditions, a limitation was observed with the 3D method. During acquisition, the Butterfly iQ device occasionally displayed a warning message stating “Bladder extends off view” (Figure 3). This occurred in 7 participants (13% of the cohort), all of whom had large bladder volumes (≥700 mL) or elongated bladder shapes. The issue is likely related to constraints in the probe’s electronic beam steering capabilities, which can limit capturing the full field of view. Despite this warning, the device continued to generate numerical volume estimates, and exclusion of these cases did not materially change the overall accuracy or reproducibility results.

Despite this, both operators noted the ease of use and efficiency of the handheld 3D device where it provided automated volume estimation by simply positioning the probe at the center of the bladder, with minimal operator input. Overall, both systems generated volume readings promptly, though the 3D method was consistently faster in acquisition.

## 4. Discussion

This study demonstrates that the automated 3D method using the handheld Butterfly iQ ultrasound device provides higher accuracy compared to the standard 2D method for bladder volume estimation. The 3D method achieved substantially improved accuracy, with percentage errors of 11–12% compared to 28–37% for the 2D method across both operators. However, both ultrasound methods systematically underestimated bladder volume compared to the reference standard; however, the 3D technique exhibited substantially less bias, with mean differences of 65–72 mL compared to 137–192 mL for the 2D method.

Both methods demonstrated excellent intra-operator reproducibility, with ICC values exceeding 0.96, indicating that individual operators can achieve highly consistent measurements with minimal training. However, the 3D method showed superior inter-operator agreement (ICC: 0.977 vs. 0.927) and markedly reduced systematic bias between operators (7.3 mL vs. 54.2 mL difference). This improved consistency across different users represents a significant advantage for clinical implementation, as it reduces operator-dependent variability that can compromise measurement reliability in practice. The study also revealed that both methods were technically feasible with 100% successful completion rates. However, the 3D method demonstrated faster acquisition times and required minimal operator input beyond probe positioning, while the 2D method required manual caliper placement and dimensional measurements.

One of the notable observations we found was the 3D method limitations with large bladder volumes (≥700 mL), where “bladder extends off view” warnings indicated field-of-view constraints. The literature supports our finding that bladder volume estimation is influenced by bladder size and shape, though most studies focus on accuracy rather than technical limitations. Vinod et al. demonstrated that both 3D ultrasound and BladderScan significantly underestimated bladder volumes, with 3D ultrasound showing a 30.1% error rate that improved to 20.7% after applying correction factors [13]. They also found that large, irregularly shaped bladders also produced greater underestimation errors. Bih et al. reported that bladder shape significantly affects volume estimation accuracy, with different correction coefficients needed for cuboidal (0.89), ellipsoid (0.81), and triangular prism-shaped (0.66) bladders [24]. The field-of-view constraint we observed likely represents a technical limitation of the Butterfly iQ handheld ultrasound device when encountering extremely distended bladders, which may have more irregular shapes that exceed the electronic beam steering capabilities. This suggests that while 3D automated methods offer superior accuracy for most clinical scenarios, alternative techniques may be necessary for patients with very large bladder capacities, particularly those with neurogenic bladder dysfunction who are more likely to develop both large volumes and irregular shapes.

Very few studies have investigated the semi-automated Butterfly iQ technology for urinary bladder volume assessment, and most have been conducted on a smaller sample size or in settings or populations different from the present work [15,16,17,18]. In an emergency department setting, Ho-Gotshall et al. compared a nursing bladder scanner, cart-based ultrasound, and Butterfly iQ against post-measurement catheterization and found that the cart-based ultrasound demonstrated the highest agreement with the gold standard, whereas Butterfly significantly overestimated catheterized volume; nonetheless, both the nursing scanner and Butterfly were rated more convenient than the cart-based system [18]. This divergence from our results likely reflects methodological differences—specifically, their use of catheterization in older, acutely unwell patients versus our use of voided volume in healthy young adults, as well as differing operator experience levels and timing of measurements, all of which can influence bias and variability.

From an implementation perspective, Nunan et al. reported that after a 20 min teaching session, ward staff confidence in using the Butterfly semi-automated visual method was significantly greater than when using automated non-visual bladder scanners, and that overall uptake was high [17]. Moreover, Jalfon et al. examined both operator- and patient-acquired postvoid residual measurements using Butterfly iQ and found excellent repeatability (ICCs 0.95–0.98) for Butterfly and standard scanners alike [15]. This aligns closely with our observation that even novice operators achieved excellent reproducibility with minimal training. The visual confirmation inherent in the Butterfly system likely reduces gross targeting errors and increases user trust, which may help explain the consistency of repeated measures observed in our cohort. Jalfon et al. also reported that Bland–Altman limits of agreement between devices exceeded their pre-specified ±50 mL threshold [15]. We found a similar pattern, where high ICC values were accompanied by wide limits of agreement, highlighting that good reliability does not necessarily equate to close absolute agreement at the individual-measurement level. The observed limits, exceeding 100–200 mL in some cases, may be clinically significant, particularly around common thresholds for catheterization (300–500 mL) or postoperative monitoring. While the handheld 3D method reduced systematic bias and improved reproducibility compared to 2D, the wide limits suggest that results should be interpreted with caution in borderline cases. We therefore recommend handheld 3D ultrasound as a more reliable adjunct to conventional methods, but not as a sole determinant when precise volume thresholds are critical to management.

In another study, Wright et al. compared Butterfly iQ, Clarius C3, and a dedicated bladder scanner for prostate and bladder volumes and found that in the bladder subset, ICCs for voided volume prediction were better for Butterfly iQ (0.82) compared to Clarius (0.72) and the bladder scanner (0.69); they also reported shorter scan times and lower device cost for Butterfly [16]. This reliability agrees with our reproducibility results and supports our feasibility findings, where both operators noted the simplicity and speed of acquisition with the 3D method. Taken together, the emerging literature suggests that Butterfly’s visual and semi-automated 3D acquisition is feasible, fast, and associated with high reproducibility. In contrast, the conventional 2D method requires manual determination of 6 calipers to calculate the volume. It could be challenging for operators with minimal sonographic skills to confidently determine the exact location of each measurement [12,25,26].

Our results have significant clinical implications. The accuracy differences observed between the two methods can directly influence bladder management decisions. The 28–37% underestimation by the 2D ultrasound method could lead to misclassification of bladder filling states, potentially resulting in inappropriate catheterization, inadequate bladder training protocols, or misinterpretation of post-void residual volumes. In contrast, the 11–12% systematic error of the 3D method approaches clinically acceptable thresholds, although the presence of systematic underestimation still warrants consideration in volume-dependent clinical interventions. Similar findings have been reported in previous comparative studies of 2D and 3D ultrasound, where 3D approaches consistently reduced error rates and improved agreement with true bladder volumes [26].

The improved inter-operator consistency of the 3D method addresses a critical limitation in point-of-care ultrasound applications. In clinical settings where multiple healthcare providers may perform bladder assessments, the reduced operator-dependent variability (7.3 mL vs. 54.2 mL systematic difference) could enhance measurement standardization and reduce training requirements. Prior research has emphasized that operator skill level and caliper placement in 2D methods are major contributors to variability [26]. Coelho et al. developed a useful and validated 23 items training checklist to assess the skill of nurses in measuring UB volume using POCUS [27]. This is particularly relevant for emergency departments, urology clinics, and nursing units where rapid, reliable bladder volume assessment is essential for patient care decisions.

The automated acquisition process of the 3D method offers additional clinical benefits by reducing training requirements and measurement time. Minimal user input is required beyond correct probe placement, which could facilitate broader implementation in resource-limited settings or situations where sonographic expertise is scarce. Other authors have suggested that such automated approaches could mitigate some of the pitfalls of conventional non-visual bladder scanners, which may misinterpret adjacent pelvic or abdominal fluid collections as bladder contents [4,26]. Nevertheless, our observation of field-of-view limitations in cases of extreme bladder distension (>700 mL) aligns with reports of incomplete volume capture in both handheld and stationary 3D systems [11,13], indicating that hybrid imaging strategies may be required for optimal accuracy across all clinical scenarios. Beyond technical performance, considerations of cost and workflow are also important. Handheld 3D ultrasound devices are generally more affordable than conventional cart-based systems and bladder scanners, while offering multipurpose imaging capabilities. Combined with faster, semi-automated acquisition, these features suggest practical advantages in clinical workflows. Nonetheless, formal cost-effectiveness studies are warranted across diverse healthcare settings.

Finally, the systematic underestimation identified in both methods—more pronounced with the 2D technique—highlights the potential role of calibration factors or correction algorithms for precise volume estimation. Similar recommendations have been made in prior work evaluating correction coefficients for bladder scanners and 3D ultrasound devices [13]. By understanding and adjusting for these biases, clinicians can interpret measurements more accurately, particularly in contexts where precise thresholds guide intervention, such as postoperative bladder monitoring, urinary retention diagnosis, or pediatric bladder management.

Our study adds several original contributions to the literature on bladder volume assessment. To our knowledge, it is the first to directly compare the Butterfly 3D method with the conventional 2D approach in the same cohort, using true voided urine volume as the reference. Unlike prior feasibility studies, we also incorporated systematic intra- and inter-operator reproducibility testing, which provides novel insight into consistency across novice users. These aspects set our work apart and support its relevance for clinical practice. The study included only novice operators. This choice was intentional to evaluate usability in real-world contexts, where bladder volume assessments are often performed by non-specialists. Despite this, intra- and inter-operator reproducibility exceeded 0.96, underscoring that the handheld 3D system can provide reliable results even with minimal training. Future research could also include experienced sonographers to examine potential performance differences across training levels. Nevertheless, our work has several limitations. First, bladder filling during the course of repeated measurements is an inherent challenge. The rate of filling varies substantially between individuals and can be influenced by factors such as hydration status, fluid intake timing, renal function, and detrusor muscle activity [2]. We assumed a constant bladder volume between scans; however, in reality, some degree of filling likely occurred between repeated measurements. Given that the acquisition time for all scans was short, no adjustments for filling rate were applied, as the potential change in volume during this window was considered negligible. Second, our sample did not include elderly participants or individuals with known bladder pathology. Older adults often have more irregular bladder morphology and thicker bladder walls due to chronic conditions such as outlet obstruction, which can make the measurements more challenging. In such cases, the semi-automated 3D acquisition may offer greater advantages over conventional techniques, but this hypothesis requires direct investigation. Although catheterization remains the most definitive reference standard, we used voided volume for its non-invasiveness. Post-void residuals were measured and found to be minimal (average <25 mL), and accounting for them did not alter the study conclusions. These small volumes may also reflect bladder refilling during repeated scans. Nonetheless, future studies in clinical populations should consider catheterization when performed as part of routine care to fully eliminate residual urine bias. Finally, the study was conducted only on healthy young male volunteers. Although both genders were invited to participate, only male students volunteered due to cultural considerations, as the scanning procedure can be slightly revealing and was conducted by male sonographers. The gender limitation may limit the generalizability of the findings to other populations, including females, pediatric patients, and those in acute or postoperative settings.

Future studies should expand to include elderly participants, females, and patients with bladder pathology (e.g., neurogenic bladder, outlet obstruction, pelvic organ prolapse), as these groups represent the populations where bladder volume measurement is most clinically relevant. Beyond accuracy, practical considerations also influence adoption. Handheld 3D ultrasound devices are portable, require minimal training compared to conventional ultrasound, and can be more readily integrated into bedside workflows. Their multipurpose use may further support cost-effectiveness, although formal health-economic analyses are warranted. To consolidate the evidence base, multicenter studies with diverse populations and systematic reviews or meta-analyses of handheld devices are also needed to confirm generalizability and guide clinical adoption.

## 5. Conclusions

In this study, the handheld Butterfly iQ automated 3D bladder volume measurement method demonstrated superior accuracy and inter-operator reproducibility compared to the conventional 2D approach in healthy young adults. The 3D method offered faster acquisition, required minimal operator input, and showed reduced variability between users, supporting its potential for broader clinical adoption, particularly in settings with variable sonographic expertise. However, both techniques showed systematic underestimation to the true voided volume. These findings highlight the value of visual, semi-automated 3D acquisition for improving standardization in bladder volume assessment and the need for further research in elderly populations, and patients with complex bladder morphologies, and in diverse clinical contexts. Additionally, future research should explore cost, workflow integration, and training implications.

## Figures and Tables

**Table 1:** Summary of Bladder Volume Estimates and Percentage Differences Compared to True Voided Volume Across Methods and Operators.

Method of Measurement		Median Volume (mL)	95% CI of the Median	IQR (mL)	Mean Difference to Voided Volume (SD)	Percentage of Difference to Void (%)
Operator A	Standard method	309.7	257.3–352.0	208.8	−191.6 (88.3)	−36.7
	3D method	436.0	394.5–524.0	236.0	−64.9 (83.7)	−11.2
Operator B	Standard method	349.8	306.8–403.4	254.6	−137.4 (69.4)	−27.6
	3D method	430.0	381.0–520.5	257.5	−72.3 (90.6)	−12.0
True voided volume		485.0	441.0–580.0	329.4	-	-

CI: Confidence interval; IQR = Interquartile range; SD = Standard deviation; 3D = 3-dimensional.

**Table 2:** Bland–Altman Analysis of Bladder Volume Estimation Methods Compared to True Voided Volume.

Method	Mean Difference (Bias)	95% CI of Bias	LoA Upper (Mean + 1.96 SD)	95% CI of Upper LoA	LoA Lower (Mean—1.96 SD)	95% CI of Lower LoA
Operator A—Standard	−191.6 mL	−216.0 to −167.3	−18.4	−60.6 to 23.8	−364.9	−407.1 to −322.7
Operator A—3D method	−64.9 mL	−88.0 to −41.9	99.1	59.2 to 139.1	−229.1	−269.0 to −189.1
Operator B—Standard	−137.4 mL	−156.6 to −118.3	−1.3	−34.5 to 31.9	−273.5	−306.7 to −240.4
Operator B—3D method	−72.3 mL	−97.3 to −47.3	105.5	62.2 to 148.8	−250.0	−293.3 to −206.7

LoA = Limits of Agreement; ICC = Intraclass Correlation Coefficient; SD = Standard deviation.

**Table 3:** Intra- and Inter-Operator Reproducibility of Bladder Volume Measurements Using Standard and 3D Ultrasound Methods.

Comparison Type	Method	ICC	95% CI of ICC	Mean Difference (Bias)	95% CI of Bias	Limits of Agreement (LoA)	95% CI of LoA
Intra-operator (Operator A)	Standard	0.977	0.960–0.986	−3.8 mL	−13.3 to 5.7	−71.2 to 63.6	−87.7 to −54.8/47.2 to 80.0
	3D	0.976	0.959–0.986	−0.3 mL	−11.4 to 10.8	−79.2 to 78.7	−98.5 to −60.0/59.5 to 97.9
Intra-operator (Operator B)	Standard	0.962	0.934–0.978	−4.8 mL	−20.0 to 10.4	−112.9 to 103.3	−139.2 to −86.6/76.9 to 129.6
	3D	0.983	0.971–0.990	5.8 mL	−2.9 to 14.6	−56.7 to 68.4	−71.9 to −41.4/53.1 to 83.6
Inter-operator (A vs. B)	Standard	0.927	0.769–0.968	−54.2 mL	−75.9 to −32.6	−208.3 to 99.9	−245.9 to −170.8/62.3 to 137.4
	3D	0.977	0.961–0.987	7.3 mL	−7.2 to 21.8	−95.6 to 110.2	−120.7 to −70.5/85.1 to 135.3

LoA = Limits of Agreement; ICC = Intraclass Correlation Coefficient; SD = Standard deviation.

**Table 4:** Agreement Between Standard and 3D Ultrasound Methods for Bladder Volume Estimation.

Comparison Type	Comparison	ICC	95% CI of ICC	Mean Difference (Bias)	95% CI of Bias	Limits of Agreement (LoA)	95% CI of LoA
Inter-method (within operator)	Operator A: Standard vs. 3D	0.798	0.599–0.936	−126.7 mL	−152.5 to −100.9	−310.3 to 56.9	−355.0 to −265.5/12.2 to 101.6
	Operator B: Standard vs. 3D	0.890	0.699–0.950	−65.2 mL	−93.0 to −37.4	−262.8 to 132.5	−311.0 to −214.7/84.4 to 180.7
Inter-method (across operators)	Operator A Standard vs. Operator B 3D	0.735	0.543–0.847	−119.4 mL	−149.1 to −89.6	−331.0 to 92.2	−382.6 to −279.5/40.7 to 143.8
	Operator A 3D vs. Operator B Standard	0.901	0.829–0.943	72.5 mL	47.5 to 97.4	−105.0 to 249.9	−148.2 to −61.8/206.7 to 293.1

LoA = Limits of Agreement; ICC = Intraclass Correlation Coefficient; 3D = 3-dimensional.

## Data Availability

The data that support the findings of this study are available from the corresponding author upon reasonable request.

## Supplementary Materials

The following supporting information can be downloaded at: https://www.mdpi.com/article/10.3390/diagnostics15172229/s1, Figure S1: Bland–Altman plots assessing intra- and inter-operator reproducibility of urinary bladder volume estimations.; Figure S2: Bland–Altman plots assessing the agreement between standard and 3D ultrasound methods for urinary bladder volume estimation.

## Abbreviations

The following abbreviations are used in this manuscript:

UB	Urinary Bladder
CI	Confidence Interval
2D	Two-dimensional
3D	Three-dimensional
ICC	Intraclass correlation coefficients
POCUS	point-of-care ultrasound

## References

[1] Segura-Grau A., Salcedo-Joven I., Montes-Belloso E., Cinza-Sanjurjo S., Segura-Fragoso A., Segura-Grau E. (2025). Usefulness of Handheld Ultrasound Devices in the Assessment of Abdominal Pathology and Comparison with High-End Ultrasound Devices. Ultrasound J..

[2] Rosier P.F.W.M., Schaefer W., Lose G., Goldman H.B., Guralnick M., Eustice S., Dickinson T., Hashim H. (2017). International Continence Society Good Urodynamic Practices and Terms 2016: Urodynamics, Uroflowmetry, Cystometry, and Pressure-Flow Study. Neurourol. Urodyn..

[3] Baldini G., Bagry H., Aprikian A., Carli F., Warner D.S., Warner M.A. (2009). Postoperative Urinary Retention: Anesthetic and Perioperative Considerations. Anesthesiology.

[4] Sullivan R., Baston C.M. (2019). When Not to Trust the Bladder Scanner. The Use of Point-of-Care Ultrasound to Estimate Urinary Bladder Volume. Ann. Am. Thorac. Soc..

[5] Bevan C., Buntsma D., Stock A., Griffiths T., Donath S., Babl F.E. (2011). Assessing Bladder Volumes in Young Children Prior to Instrumentation: Accuracy of an Automated Ultrasound Device Compared to Real-Time Ultrasound. Acad. Emerg. Med..

[6] Beckers G.M., Van Der Horst H.J.R., Frantzen J., Heymans M.W. (2013). The BladderScan BVI 6200^®^ Is Not Accurate Enough for Use in a Bladder Retraining Program. J. Pediatr. Urol..

[7] Zheng V.J., Geynisman-Tan J., Knoll J., Kenton K., Brown O. (2023). Accuracy of Bladder Scanner in Measuring Bladder Volumes in Postpartum Women. Urogynecology.

[8] Alnaif B., Drutz H.P. (1999). The Accuracy of Portable Abdominal Ultrasound Equipment in Measuring Postvoid Residual Volume. Int. Urogynecology J..

[9] Theisen J.G., Deveneau N.E., Agrawal A., Kinman C., Gaskins J., Meriwether K., Francis S.L. (2019). The Accuracy of Portable Ultrasound Bladder Scanner Measurements of Postvoid Residual Volume in Women with Pelvic Organ Prolapse. Urogynecology.

[10] Liang C.C., Wei T.Y., Chang S.D., Hsieh C.C. (2009). Bladder Volume Determination: Two-Dimensional Versus Three-Dimensional Transvaginal Ultrasound. Taiwan J. Obstet. Gynecol..

[11] Chang M.L., Li H.C., Liu C.K., Chiang H.S., Hsu C.C. (2021). Novel Three-Dimensional Bladder Reconstruction Model from b-Mode Ultrasound Image to Improve the Accuracy of Bladder Volume Measurement. Sensors.

[12] Nagle A.S., Bernardo R.J., Varghese J., Carucci L.R., Klausner A.P., Speich J.E. (2018). Comparison of 2D and 3D Ultrasound Methods to Measure Serial Bladder Volumes During Filling: Steps Toward Development of Non-Invasive Ultrasound Urodynamics. Bladder.

[13] Vinod N.N., Nagle A.S., Naimi H.A., Kolli H., Sheen D., Nandanan N., Carucci L.R., Speich J.E., Klausner A.P. (2019). Bladder Volume Correction Factors Measured with 3D Ultrasound and BladderScan. Can. J. Urol..

[14] Ghani K.R., Pilcher J., Rowland D., Patel U., Nassiri D., Anson K. (2008). Portable Ultrasonography and Bladder Volume Accuracy-A Comparative Study Using Three-Dimensional Ultrasonography. Urology.

[15] Jalfon M., Gardezi M., Heckscher D., Shaheen D., Maciejewski K.R., Li F., Rickey L., Foster H., Cavallo J.A. (2024). Agreement and Reliability of Patient-Measured Postvoid Residual Bladder Volumes. Urology.

[16] Wright H.C., Corrigan D., De S. (2024). Can Handheld Ultrasound Probes Reliably Measure Transabdominal Prostate and Bladder Volumes? A Prospective Randomized Point-of-Care Ultrasound Study. Front. Urol..

[17] Nunan J., Lister T., Howgill H., Marie M., Parreno M., Brown G., Walden A. (2024). Point of Care Ultrasound Bladder Volume Calculation on the Acute Medical Unit. Acute Med..

[18] Ho-Gotshall S., Wilson C., Jacks E., Kashyap R. (2024). Handheld Ultrasound Bladder Volume Assessment Compared to Standard Technique. Cureus.

[19] Bizzarri F.P., Campetella M., Ragonese M., Scarciglia E., Russo P., Marino F., Filomena G.B., Gavi F., Rossi F., Sacco E. (2024). The Role of Alternative Medicine and Complimentary Therapies in Urologic Disease: New Horizons. Urol. J..

[20] Alfuraih A.M., Alrashed A.I., Almazyad S.O., Alsaadi M.J. (2021). Abdominal Aorta Measurements by a Handheld Ultrasound Device Compared with a Conventional Cart-Based Ultrasound Machine. Ann. Saudi Med..

[21] Alfuraih A.M., Alqarni M.A., Alhuthaili H.S., Mubaraki M.Y., Alotaibi N.N., Almusalim F.M. (2023). Reproducibility and Feasibility of a Handheld Ultrasound Device Compared to a Standard Ultrasound Machine in Muscle Thickness Measurements. Australas. J. Ultrasound Med..

[22] Butterfly Network Inc IQ+ Bladder Technical Details. https://www.butterflynetwork.com/iq-bladder-specs?srsltid=AfmBOorHybnjhMUv2cMWPhNeB3XgLJ8IhUhbS0gOJTNeefrNvTy_w9eG.

[23] JASP Team (2025). JASP.

[24] Bih L.-I., Ho C.-C., Tsai S.-J., Lai Y.-C., Chow W. (1998). Bladder Shape Impact on the Accuracy of Ultrasonic Estimation of Bladder Volume. Arch. Phys. Med. Rehabil..

[25] Alpert E.A., Gold D.D., Kobliner-Friedman D., Wagner M., Dadon Z. (2024). Revolutionizing Bladder Health: Artificial-Intelligence-Powered Automatic Measurement of Bladder Volume Using Two-Dimensional Ultrasound. Diagnostics.

[26] Schallom M., Prentice D., Sona C., Vyers K., Arroyo C., Wessman B., Ablordeppey E. (2020). Accuracy of Measuring Bladder Volumes with Ultrasound and Bladder Scanning. Am. J. Crit. Care.

[27] Coelho F.U.d.A., Reigota S.M., Cavalcanti F.M., Regagnin D.A., Murakami B.M., Santos V.B. (2024). Bladder Ultrasound: Evidence of Content Validity of a Checklist for Training Nurses. Rev. Bras. Enferm..

