# Supplementary material for: Accuracy and Reproducibility of Handheld 3D Ultrasound Versus Conventional 2D Ultrasound for Urinary Bladder Volume Measurement: A Prospective Comparative Study
Source: Diagnostics (Basel). 2025 Sep 3;15(17):2229. doi: 10.3390/diagnostics15172229 (PMC12428280; doi:10.3390/diagnostics15172229)
Supplement: Supplementary file 1 [file diagnostics-15-02229-s001.zip › diagnostics-3847585-supplementary.pdf]

## Supplementary material

**Figure S1. Bland-Altman plots assessing intra- and inter-operator reproducibility of urinary bladder volume estimations.**

**(A) Operator A – standard method (first vs. second measurement),**

**(B) Operator A – 3D method (first vs. second measurement),**

**(C) Operator B – standard method (first vs. second measurement),**

**(D) Operator B – 3D method (first vs. second measurement),**

**(E) Inter-operator comparison – standard method (Operator A vs. Operator B), and**

**(F) Inter-operator comparison – 3D method (Operator A vs. Operator B).**

**Each plot displays the mean difference (bias) and the 95% limits of agreement (±1.96 SD), with narrower limits and lower bias observed for the 3D method in both intra- and inter-operator comparisons.**

**A**

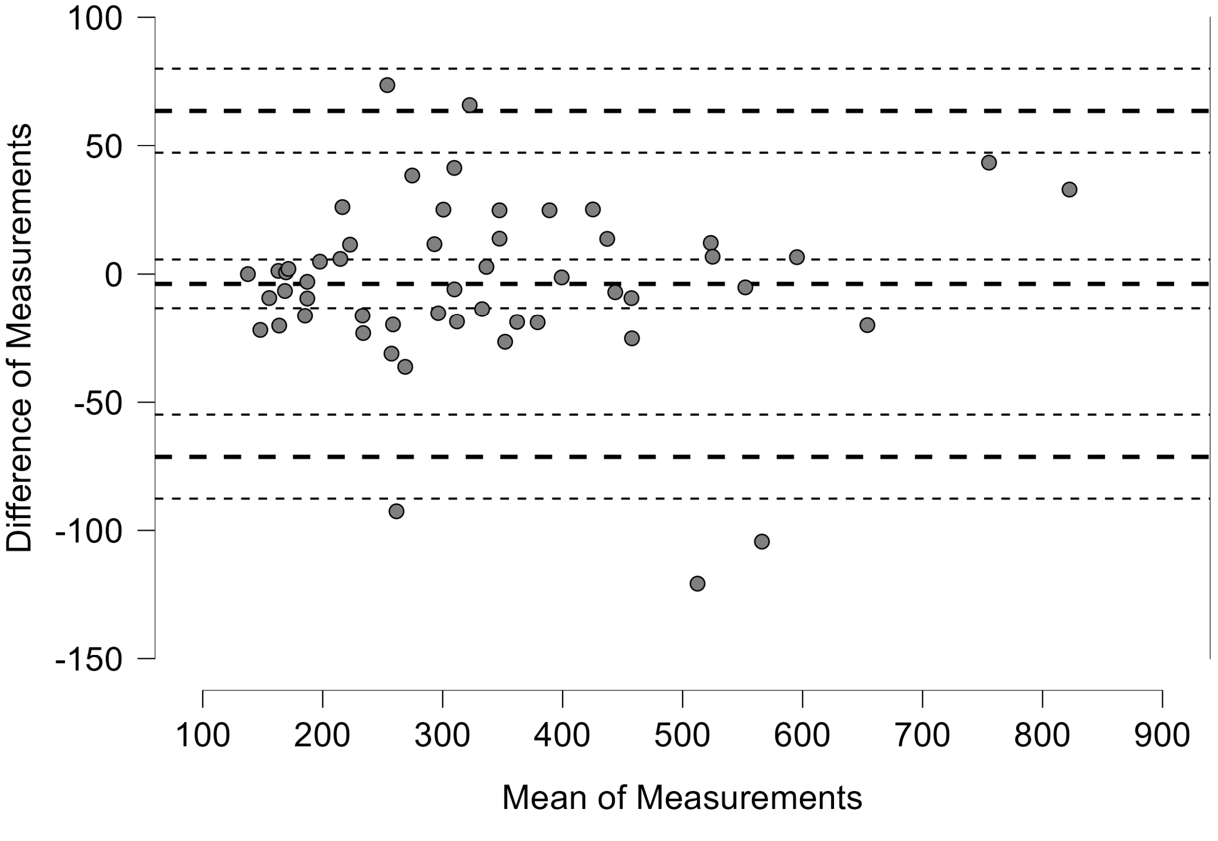

**B**

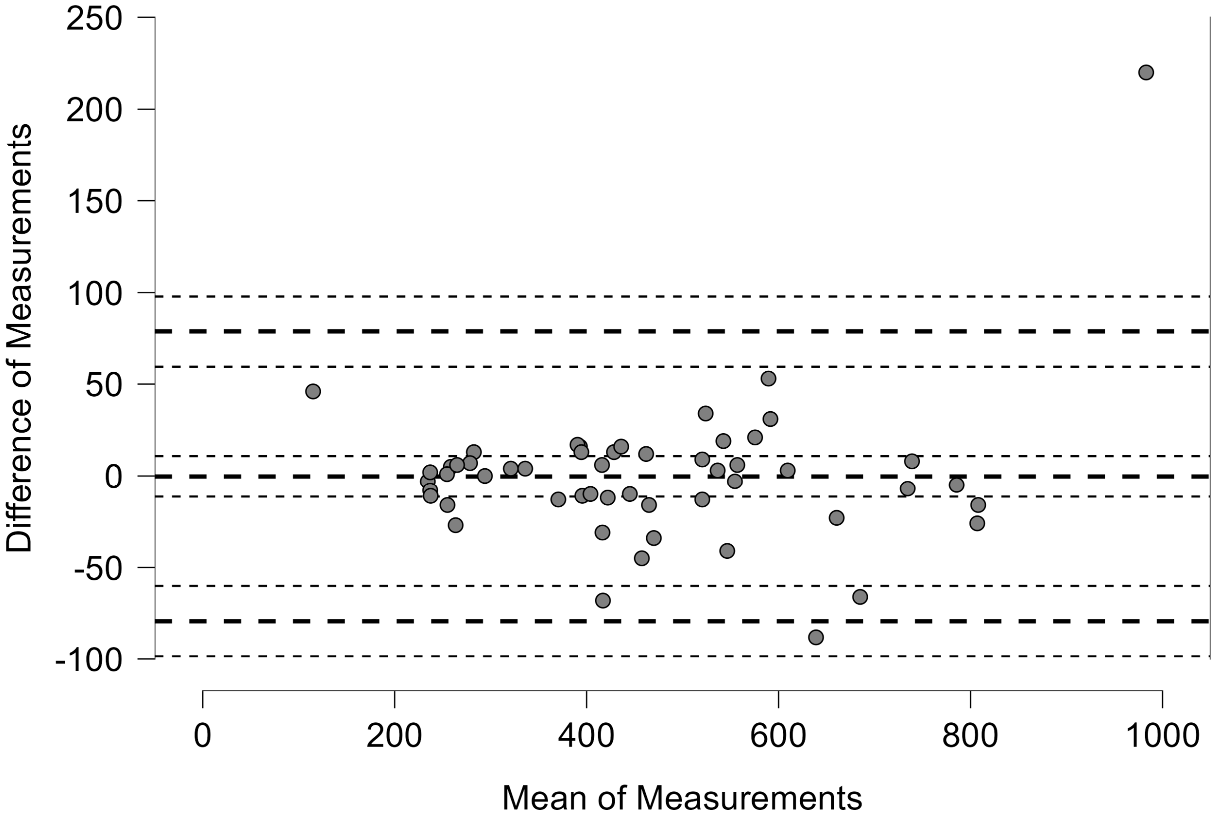

250
200
150
100
50
0
-50
-100
Difference of Measurements
0
200
400
600
800
1000
Mean of Measurements

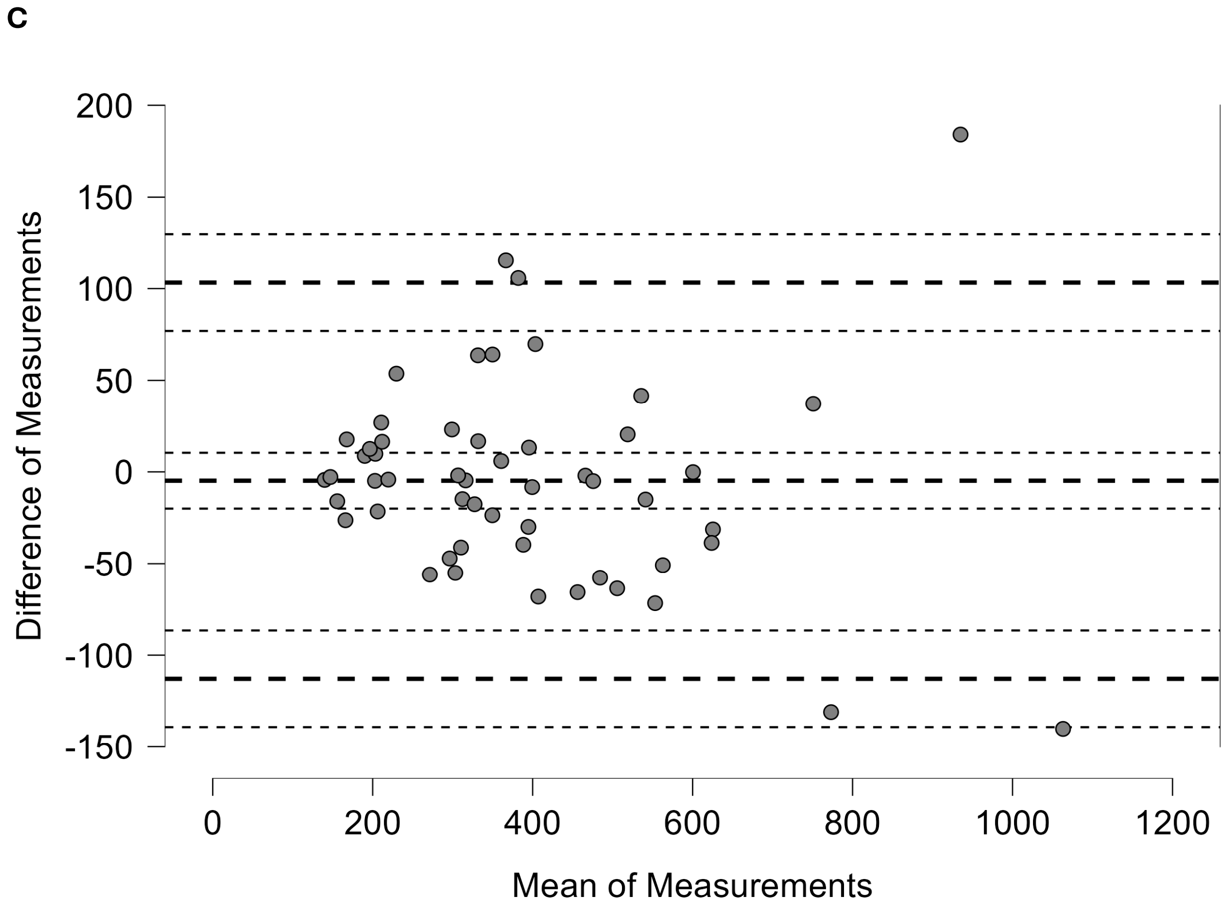
c
200
150
100
50
0
-50
-100
-150
Difference of Measurements
0
200
400
600
800
1000
1200
Mean of Measurements

**D**

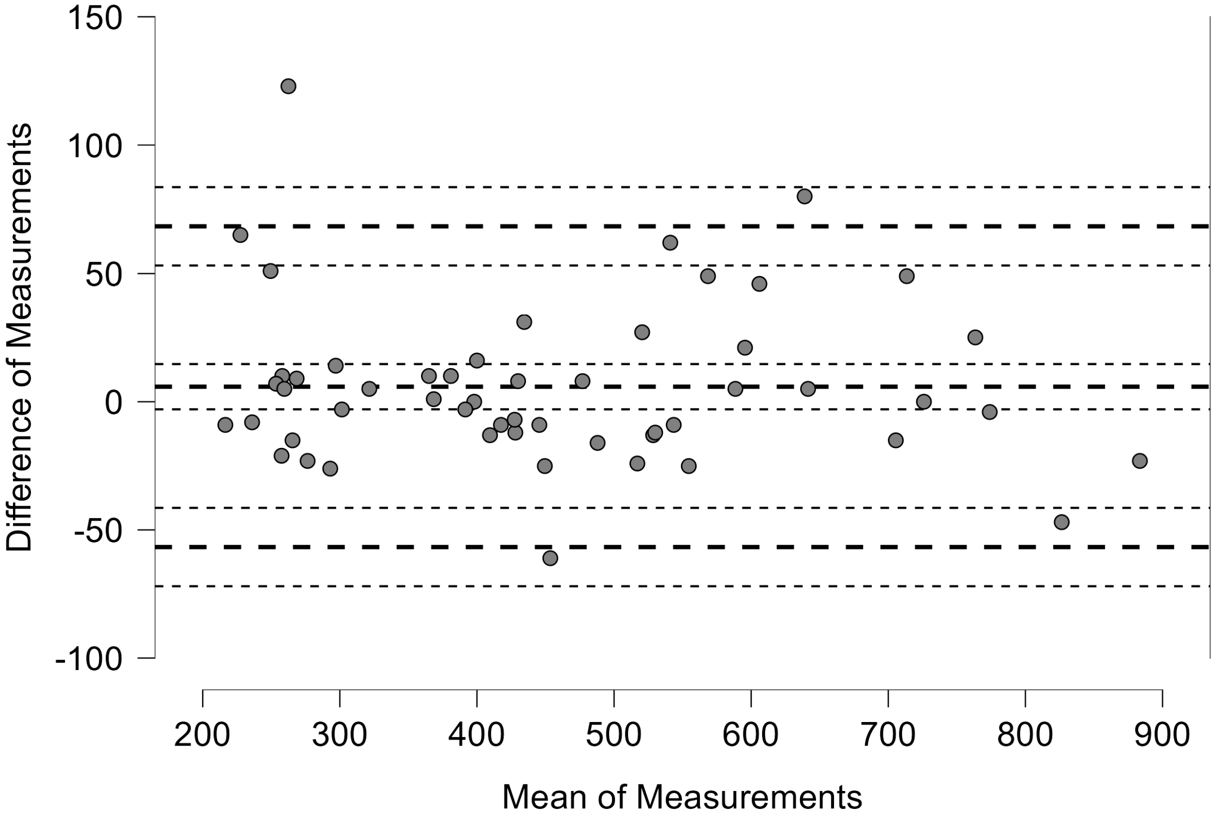
150
100
50
0
-50
-100
Difference of Measurements
200
300
400
500
600
700
800
900
Mean of Measurements

E

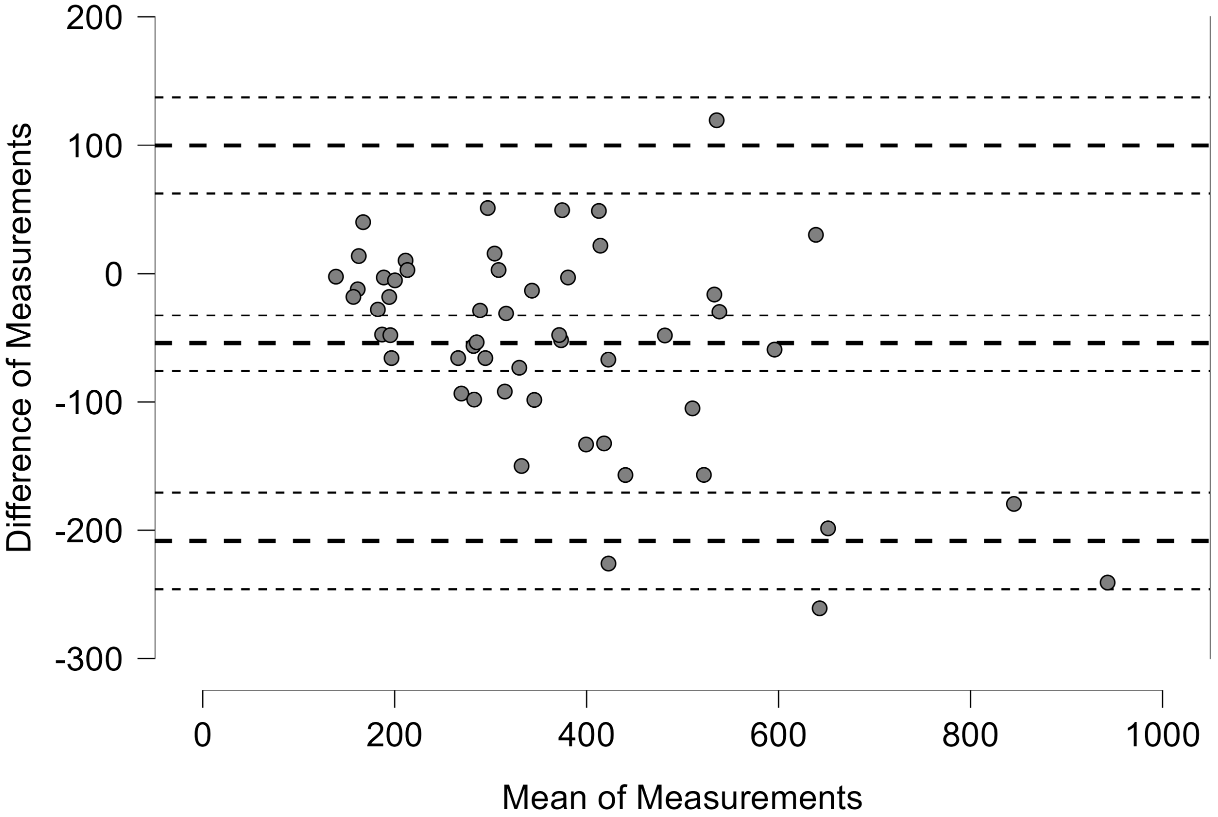

200
100
0
-100
-200
-300
Difference of Measurements
0
200
400
600
800
1000
Mean of Measurements

F

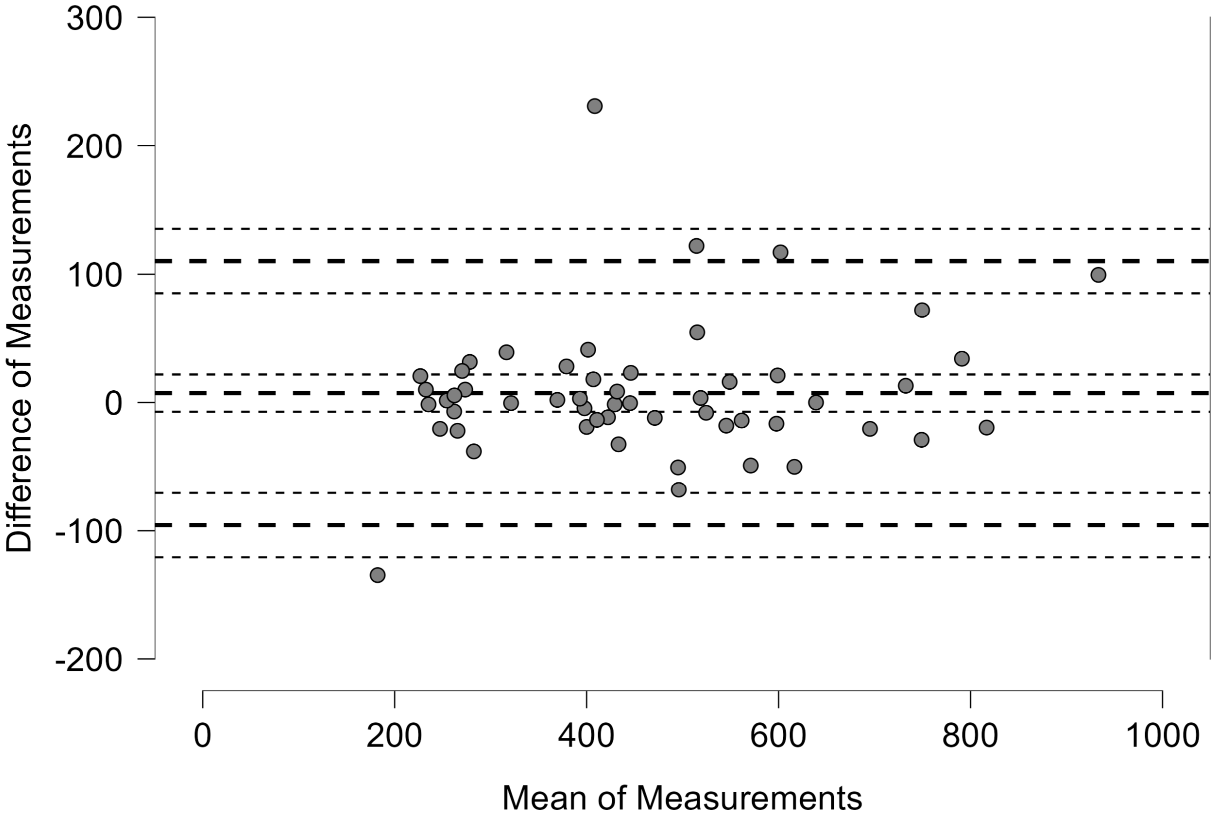

300
200
100
0
-100
-200
Difference of Measurements
0
200
400
600
800
1000
Mean of Measurements

**Figure S2. Bland-Altman plots assessing the agreement between standard and 3D ultrasound methods for urinary bladder volume estimation.**
**(A) Operator A – standard vs. 3D method,**
**(B) Operator B – standard vs. 3D method,**
**(C) Operator A standard vs. Operator B 3D method, and**
**(D) Operator A 3D method vs. Operator B standard method.**
**Each plot shows the mean difference (bias) and 95% limits of agreement (±1.96 SD). The plots highlight the presence of systematic differences between methods, with narrower limits and smaller bias generally observed when 3D measurements are compared across operators.**

**A**

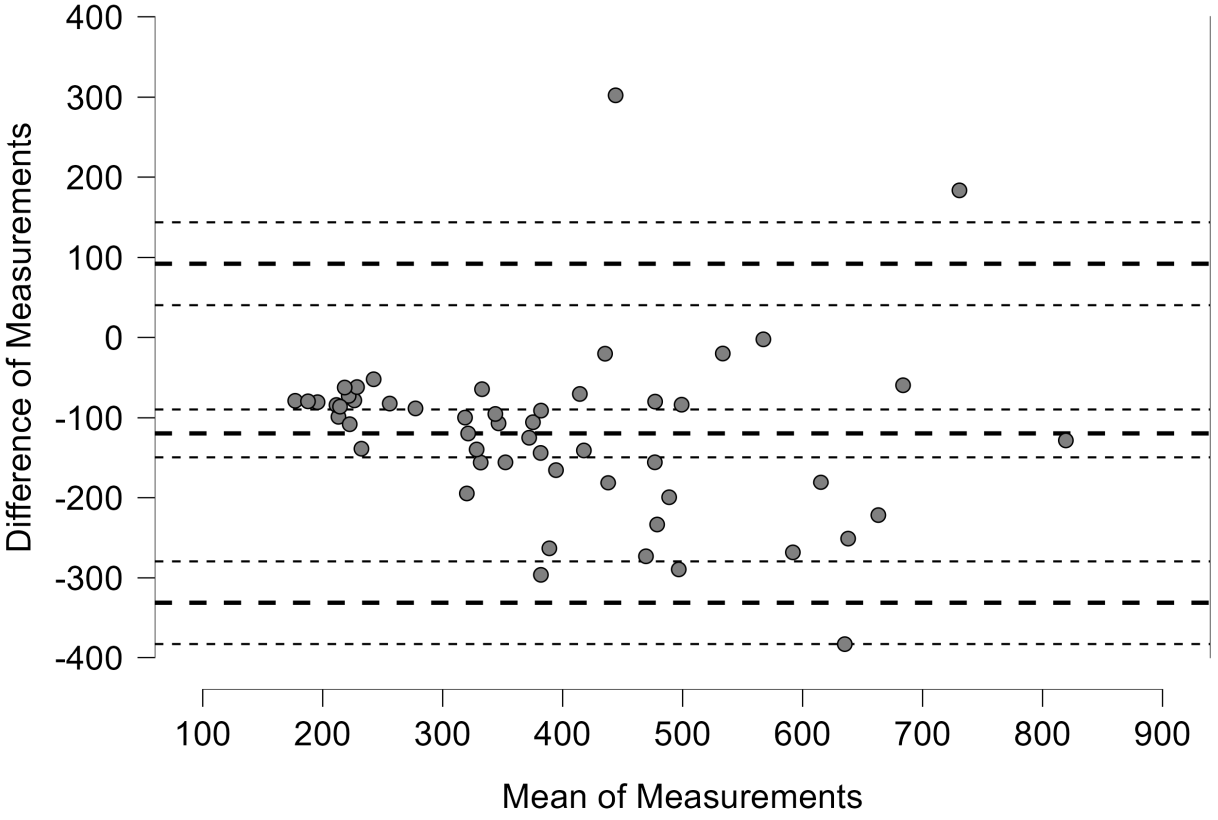

**B**

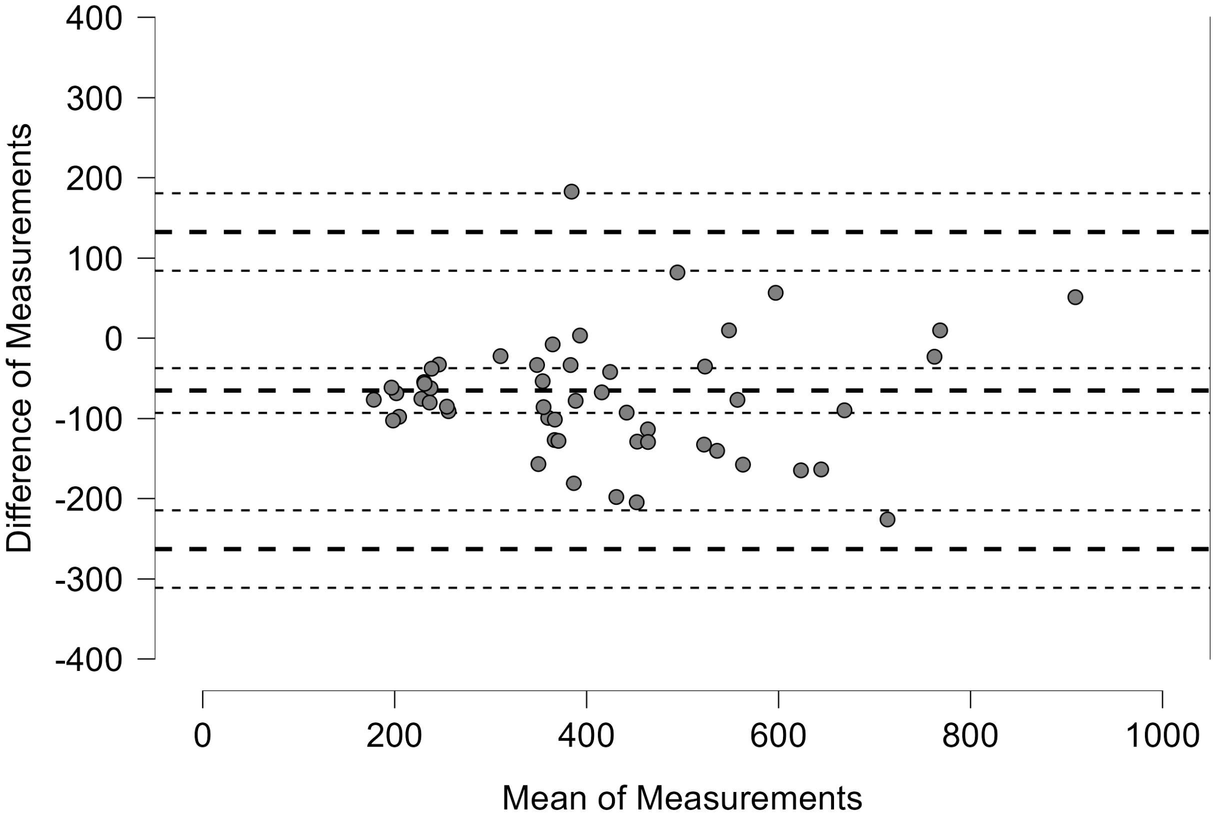

400
300
200
100
0
-100
-200
-300
-400
Difference of Measurements
0
200
400
600
800
1000
Mean of Measurements

**c**

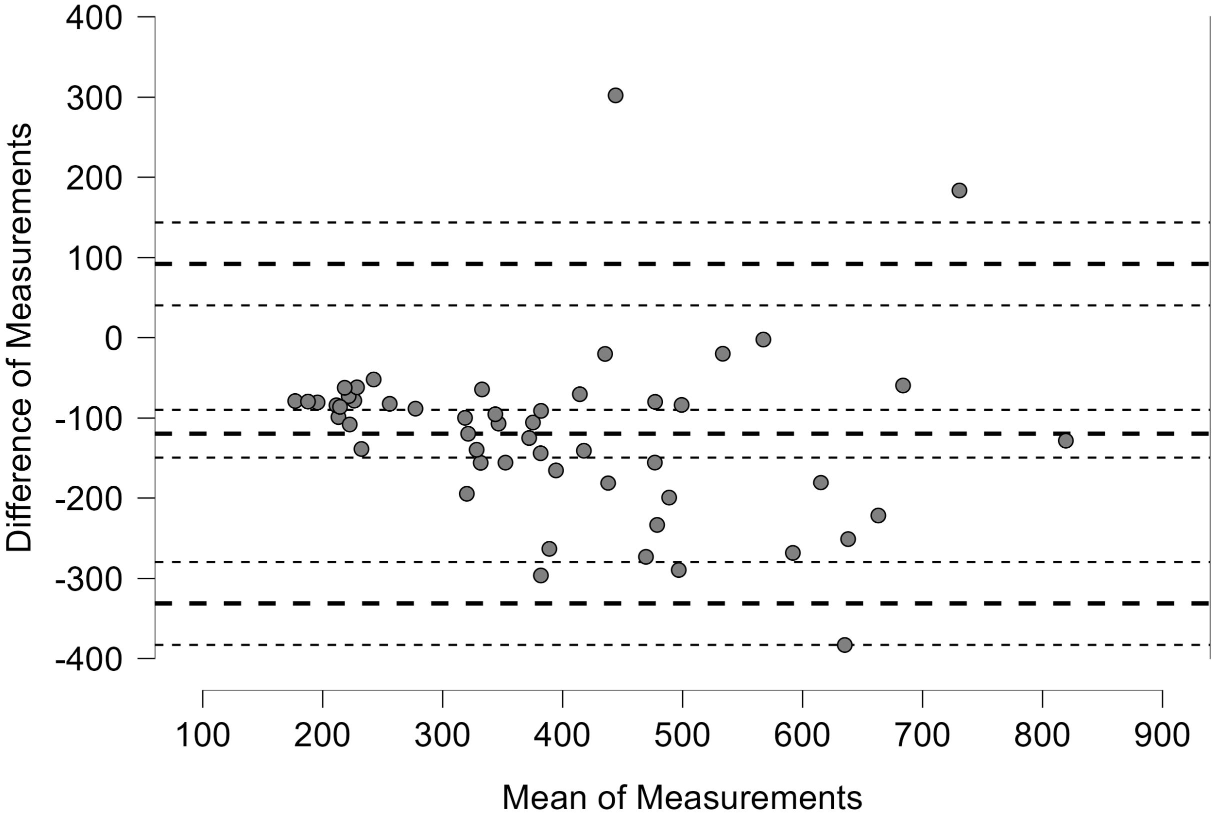

400
300
200
100
0
-100
-200
-300
-400
Difference of Measurements
100
200
300
400
500
600
700
800
900
Mean of Measurements

**D**

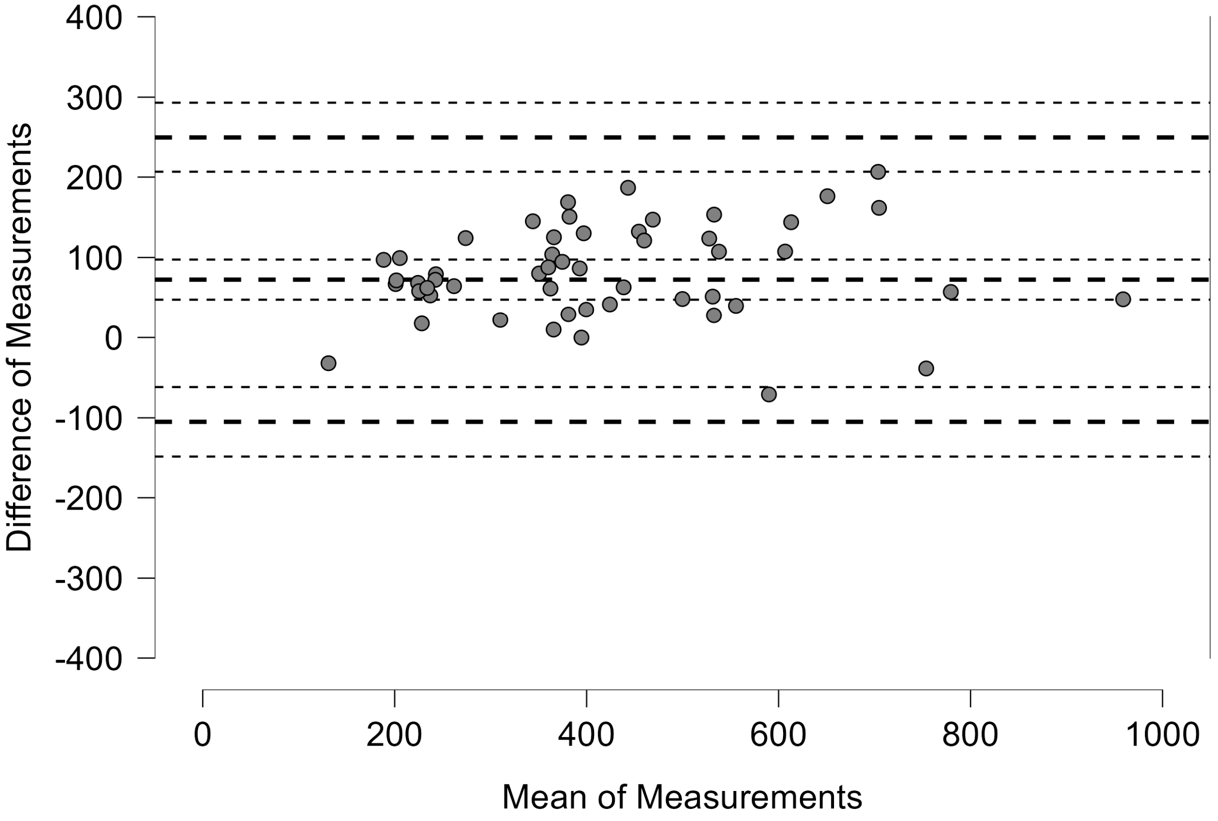
400
300
200
100
0
-100
-200
-300
-400
Difference of Measurements
0
200
400
600
800
1000
Mean of Measurements
